# Beneficial Effects of Lactic Acid Bacteria on Animal Reproduction Function

**DOI:** 10.1155/2022/4570320

**Published:** 2022-11-30

**Authors:** Mohamad Yusril Nur Mahendra, Tri Bhawono Dadi, Juriah Kamaludeen, Herinda Pertiwi

**Affiliations:** ^1^Department of Health Studies, Faculty of Vocational Studies, Airlangga University, Jalan Dharmawangsa Dalam 28-30, Surabaya 60286, Indonesia; ^2^Department of Veterinary Clinic, Faculty of Veterinary Medicine, Airlangga University, Jalan Mulyorejo, Surabaya 60115, Indonesia; ^3^Department of Animal Science and Fishery, Faculty of Agriculture and Forestry, Universiti Putra Malaysia, Bintulu Sarawak Campus, Bintulu 97008, Sarawak, Malaysia; ^4^Institute of Tropical Agriculture and Food Security, Universiti Putra Malaysia, Serdang 43400, Selangor, Malaysia

## Abstract

Considering the importance of a healthy uterus to the success of breeding, the beneficial effects of lactic acid bacteria on animal reproduction function are of particular interest. In recent decades, infertility has become a widespread issue, with microbiological variables playing a significant role. According to reports, dysbiosis of the vaginal microbiota is connected with infertility; however, the effect of the normal vaginal microbiota on infertility is unknown. In addition, lactic acid bacteria dominate the reproductive system. According to evidence, vaginal lactic acid bacteria play a crucial role in limiting the invasion of pathogenic bacteria by triggering anti-inflammatory chemicals through IL-8, IL-1, and IL-6; immunological responses through inhibition of the adherence of other microorganisms, production of inhibiting substances, and stimulation of mucus production; and also reproductive hormones by increased testosterone hormone release, enhanced the levels of luteinizing hormone, follicle stimulating hormone, the amount of prostaglandin E (2), and prostaglandin F2 alpha. The objective of this study was to compare the advantages of lactic acid bacteria in animal reproduction based on the most recent literature. The administration of a single strain or numerous strains of lactic acid bacteria has a favourable impact on steroidogenesis, gametogenesis, and animal fertility.

## 1. Introduction

Enhancing an animal's return requires high reproductive efficiency [1]. To breed regularly, animals must have functional ovaries, exhibit oestrous behaviour, mate, undergo ovulation, fertilization, conceive, support the embryo during gestation, give birth, continue oestrous cyclicity, and recover uterine function after giving birth [2]. According to Krpalkova et al. [3], infertility is a significant issue that causes economic losses and accounts for the biggest proportion of the total cost in the livestock production system. Economic losses related to infertility issues were the cost of veterinarian intervention, the predicted cost due to calf loss, the cost of cows killed, and the cost of milk loss [4].

The vagina has an inherent microbiome, and dysbiosis of vaginal microbiota or invasion by pathogens may affect fertility by directly reducing spermatozoa's motility or indirectly by producing organic injuries to the reproductive system [5]. The predominant bacterial genus in the vaginal tract is *Lactobacillus* sp. There is evidence that vaginal *Lactobacillus* sp. plays a crucial role in avoiding the invasion of pathogenic bacteria and dysbiosis of native microbiota [6]. These lactic acid bacteria's influence on fertility and their role in promoting fecundity could be discussed from two main perspectives: first, male fertility and the potential antioxidant impact of lactic acid bacteria on sperm parameters, testicular histopathology, and testosterone level and second, female fertility and the effect of lactic acid bacteria on maintaining the bacterial balance in the vagina, treatment of bacterial vaginosis, and the subsequent effect on amelioration of bacterial vaginosis [7].

## 2. Prebiotics, Probiotics, and Synbiotics

The World Health Organization (WHO) defines “probiotics” as living microorganisms that have a positive effect on the host's health [8]. According to the descriptions provided by the International Scientific Association for Probiotics and Prebiotics (ISAPP), the range of goods that could be categorized as probiotics includes helpful bacteria and other types. These include medications and enteral feedings for disease treatment, dietary supplements for health promotion, infant formulas such as milk powders, and even animal feedings [9].

In dairy cows, the reproductive tract can be separated into an upper and lower section based on bacterial presence [10]. The top portion, which includes the fallopian tubes, uterus, and endocervix, is often devoid of bacteria, whereas the bottom portion, which includes the ectocervix and vagina, contains bacteria. Aerobic, facultatively anaerobic, and obligately anaerobic bacteria can be found in the vaginal canal of dairy cows [11]. Plate culture research reveals that the major bacteria in the vaginal canal of healthy heifers are *Enterococcus* and *Staphylococcus*, followed by *Enterobacteriaceae* and *Lactobacilli* [12]. Moreover, recent research on the uterine microbiome of cows has revealed a vast difference between healthy and metritic or endometritic cows [10]. Regardless of health status, the majority of the uterine microbiota consists of *Bacteroidetes*, *Fusobacteria*, *Firmicutes*, *Proteobacteria*, and *Tenericutes* [13]. Also, the vaginal microbiota of cows contains numerous LAB species, such as *Lactobacillus* spp., *Pediococcus* spp., *Leuconostoc* spp., and *Weissella* spp., some of which were isolated and evaluated for their probiotic powers against urogenital infections [11, 12].

Probiotics are used as feed additives because they have positive effects on animals, such as boosting the immune system [14, 15], eliminating pathogenic bacteria by preventing colonization [16], preventing infection, and enhancing the overall health of the gastrointestinal tract (GIT) [17, 18]. In addition, the results of previous studies indicate that probiotics may be an alternative to antibiotics [19].

Through competing processes, the presence of LAB may have decreased the direct interaction of pathogens with epithelial cells [20]. Otero and Nader-Macas [21] observed that *Lactobacillus* spp. isolated from the bovine vaginal tract adhered to vaginal epithelial cells at pH 4.5 and 7. Intriguingly, surface layer proteins, which are substances released by probiotic bacteria such as *L. helveticus*, can also occupy the binding sites on epithelial cells, blocking them from pathogens [22]. Such proteins serve as an epithelial surface lining, hence increasing epithelial integrity and tight junctions [23]. Due to their increased affinity for cell receptors, lactobacilli competitively exclude the adherence of pathogens to the epithelium or even displace pathogens that are already linked to the epithelium [22, 24].

**Figure 1:**
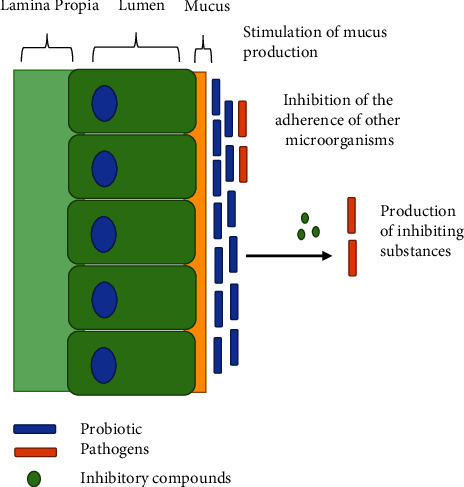
Mechanisms of pathogen inhibition by LAB probiotics.

According to Nader-Macas et al. [25], LAB strains isolated from the vaginal tract are highly capable of generating H_2_O_2_. *Lactobacilli* from the vaginal tract of calves that produce H_2_O_2_ and lactic acid have the potential to be used as probiotics, with *L. gasseri* CRL1421 having the greatest potency to inhibit *Staphylococcus aureus* [21]. A few strains of LAB (mostly *Lactobacillus fermentum*) isolated from cow vaginal mucus have been shown to inhibit the growth of *A. pyogenes* in vitro. *A. pyogenes* is a known pathogen isolated from metritic cows [26]. Through the synthesis of pediocin, *Pediococcus acidilactici* isolated from healthy pregnant dairy cows inhibits *L. innocua* and *E. faecalis* [11]. In addition, pediocin PA, a bacteriocin generated by *P. acidilactici*, has been characterized as *Listeria* and other pathogen-inactivating agents [27]. Furthermore, Ohland and Macnaughton [22] found that *lactobacilli* might boost the expression of mucin, the primary component of the mucus layer, thereby inhibiting pathogens from entering the epithelium in in vitro experiments (Figure 1.). In addition, a recent study has revealed that higher MUC3 mucin secretion reduces the adherence of *E. coli* strains [28, 29].

Some complex sugars are utilized as prebiotics to increase the likelihood of survival and persistence of bacteria in GIT [30]. Prebiotics are nondigestible compounds that, by modulating the makeup and activity of the gut microbiota, bestow a favourable physiological effect on the host [31]. Numerous substances have been evaluated to establish their prebiotic properties. The most prevalent prebiotics are fructooligosaccharides (FOSs), galactooligosaccharides (GOSs), and transgalactooligosaccharides (TOSs) [32].

The type of gut bacteria and the structure of prebiotics determine fermentation products [33]. Short-chain fatty acids (SCFAs), lactic acid, butyric acid, and propionic acid are produced by the fermentation of prebiotics by gut microbes. These products may have multiple physiological consequences. SCFAs, for instance, lower the pH of the colon [19]; propionate impacts T helper 2 in airways and macrophages, as well as dendritic cells in the bone marrow [34, 35]. Peptidoglycan is an additional prebiotic fermentation product that can enter the bloodstream and stimulate the innate immune system against pathogenic bacteria [35].

Synbiotics combine probiotics and prebiotics synergistically [36]. Understanding problems of sexual differentiation, reproduction, fertility, hypertension, obesity, and physiologic equilibrium, a synbiotic product has a positive effect on the host by enhancing the survival and implantation of live microbial dietary supplements in the gastrointestinal system [37]. In addition, testosterone is produced by Leydig cells through steroidogenesis, which are clustered in the testicular interstitium, by selectively boosting the growth and activating the metabolism of one or a restricted number of health-promoting bacteria [38]. The administration of synbiotics has similarly favourable effects on the gut microbiota. For instance, synbiotics have been shown to increase the number of *Bifidobacteria* and *Lactobacillus*, improve stool frequency and mucosal integrity, increase butyrate production, reduce proinflammatory response, and boost lipid metabolism [39–41]. In addition, synbiotics dramatically reduced the incidence of metabolic syndrome, cardiovascular risk factors, and insulin resistance markers among aged people [42].

## 3. Role of Lactic Acid Bacteria in Steroidogenesis and Gametogenesis

Steroidogenesis is the conversion of cholesterol to glucocorticoids, mineralocorticoids, and sex steroids, which govern physiology and development [43]. Understanding steroidogenesis and its regulation is essential for signals stimulated by luteinizing hormones. For example, it has been observed that probiotic *Lactobacillus* sp. treatment can increase male reproductive organ function and testosterone hormone release. Using *Lactobacillus reuteri* ATCC 6475 at a dose of 3.5 × 10^5^ organisms per mouse per day, Poutahidis et al. [44] observed an increase in volume, core diameter, and interstitial Leydig cell area, leading to an increase in testosterone levels.

According to Baer [45], a decrease in testosterone levels results in a variety of negative effects, including a decrease in spermatogenesis, libido, and sexual function, an increase in body fat, a decrease in muscle and bone mass, low energy levels, fatigue, poor physical performance, depressed mood, and cognitive impairment. Gametogenesis is an important aspect of mammalian reproduction in which the germ cell lineage undergoes a series of complex developmental stages and produces mature gametes, spermatozoa, and oocytes [46]. In the same investigation, Poutahidis et al. [44] found that the sperm of mice treated with *L. reuteri* had significantly higher concentration and activity than age-matched control animals.

Previous studies showed that pretreatment of mice with *Lactobacillus rhamnosus G. G*. at 10^9^ CFU/ml twice daily for three consecutive days enhanced the amount of basal mucosal prostaglandin E(2) [47], whereas the presence of PGE2 could induce steroidogenesis, production of progesterone, estrone, and estradiol, steroidogenic acute regulatory protein (StAR), and cytochrome P450 family 19 subfamily A member 1 (CYP19A1 gene); this is not the case for steroidogenic acute regulatory protein (StAR) and cytochrome P450 family 19 subfamily A member 1 (CYP19A1 gene) [48]. In addition, Dardmeh et al. [49] discovered that supplementation with *Lactobacillus rhamnosus PB01* (DSM-14870) at a dose of 1 × 10^9^ CFU enhanced the levels of luteinizing hormone (L H) (2.170.22 mIU/ml) and follicle-stimulating hormone (FSH) (7.722.05 mIU/ml) in mice. During oocyte meiotic maturation, the L H surge releases oocytes from meiotic prophase arrest and encourages the continuation of oocyte meiosis and completion of the first meiotic division, enhancing oogenesis performance [50].

## 4. Implication of Lactic Acid Bacteria on Animal Fertility-Related Parameters

There is a direct connection between animal uterine health and reproduction [51]. Fertility issues in animal production units (PAUs) are known to be multifactorial, including disorders in oogenesis, oocyte degeneration, ovulation disorders, failure of fertilization, inflammation of the ovary, disorders of the oviducts, alterations in the uterus such as metritis and endometritis, and early embryonic mortality [52].

When bacterial infection causes severe or prolonged endometrial inflammation, it develops uterine disorders [53]. In addition, proinflammatory substances, such as prostaglandins and cytokines, such as interleukin 1A (IL1A), interleukin 1B (IL1B), and C-X-C motif chemokine ligand 8 (CXCL8), were elevated in cows with preclinical and clinical endometritis [54].

Previous research by Peter et al. [55] using intrauterine supplementation of *Lactobacillus buchneri* DSM 32407 in cattle demonstrated an increase in tumour necrosis factor mRNA expression (TNF). After three weeks of treatment, the endometrial mRNA expression of many proinflammatory factors (CXCL1/2, CXCL3, CXCR2, IL1B, IL8, and PTPRC) was reduced. The production of CXCL5 is induced by TNF activation of cells [56]. CXCL5 is a chemoattractant that mediates neutrophil recruitment during inflammation and infection; it binds to CXCR2, which is mostly found on the surface of immunological cells, such as polymorphonuclear (PMN) leukocytes [57]. In addition, *L. buchneri* DSM 32407 supplementation increased total PMN infiltration, indicating that cows with high PMN infiltration within the uterus have greater fertility than cows with low PMN infiltration [10].

Similar research by Gartner et al. [58] demonstrated that *Lactobacillus amylovorus* isolated from bovine endometrial epithelial cells increased prostaglandin-endoperoxide synthase 2 (PTGS2) expression. The PTGS2 gene, which encodes cyclooxygenase 2 (COX-2) in the body, will play an essential role in oocyte competence acquisition. Moreover, in research utilizing knockout mice models, the elimination of PTGS2 resulted in many reproductive failures, including ovulation, fertilization, implantation, and decidualization damage, demonstrating that prostaglandins produced by COX-2 play an essential role in reproductive processes [59].

A study conducted by Dim et al. [60] using *Lactobacillus acidophilus* NRRL-4495 (10^8^ CFU/ml) in chicks demonstrated improvement in semen quality, sperm concentration (5.82 × 10^9^/ml), progressive motility (82.93%), live sperm (94.13%), dead sperm (5.87%), normal sperm (91.38%), and abnormal sperm (8.62%). Probiotics' role in producing trace minerals and vitamins in the birds' intestines, which boosts the quality of the sperm, may be responsible for the enhanced sperm quality.

Furthermore, probiotics are associated with fish reproduction by enhancing their fecundity rate [61, 62]. Direct effects are reportedly due to increasing expression of genes, encoding several hormones and improving gonadal growth, fecundity, and embryo survival [63]. Probiotics also increase follicle maturation and development and embryo quality. For example, several strains *of Lb. rhamnosus* reported have progressive effects on accelerating the fecundity in zebrafish (*Danio rerio*) models [61].

## 5. Combinatorial Effects of Lactic Acid Bacteria (as Multistrain Probiotic) on Animal Fertility Outcomes

Multistrain lactic acid probiotics may have a larger spectrum of effects and more mechanisms of action than single-strain probiotics. For example, multistrain probiotic supplementation with *Lactobacillus sakei* FUA3089, *Pediococcus acidilactici* FUA3138, and *Pediococcus acidilactici* FUA3140, with a cell count of 10^8^–10^9^ CFU/dose, was able to produce greater concentrations of PGE2 and prostaglandin F2 alpha metabolite (PGFM) in cattle [20, 64].

The concentration of plasma PGFM is typically used to evaluate the release of endometrial PGF2 secretion once it has a longer half-life in peripheral circulation [65]. This prostaglandin F2 aids the ovulatory process and promotes optimal gamete transport, thereby enhancing fertility [66]. This multistrain probiotic is also used to lower the prevalence of uterine infections associated with elevated vaginal mucus secretory IgA (sIgA) levels [67]. Secretory IgA (SIgA) plays an important role in the protection and homeostatic regulation of intestinal, respiratory, and urogenital mucosal epithelia, separating the outside environment from the inside of the body, which is involved in preventing opportunistic pathogens from entering and disseminating in the systemic compartment, as well as tightly regulating the symbiotic relationship between commensals and the host [68].

Metritis causes infertility in multiple ways: first, by delaying the return to cyclicity after delivery; second, by disrupting the uterine environment; and third, by impeding embryo development [69, 70]. Genis et al. [71] revealed that a combination of *L. rhamnosus, P. acidilactici,* and *L. reuteri* produced by CECT (Coleccion Espanola de Cultivos Tipo, CSIC Valencia, Spain) at a ratio of 25 : 25 : 2 had the greatest capacity to modulate *E. coli* infection and secretion of inflammation markers (IL-8, IL-1, and IL-6) in vitro when compared to individual LAB strains (Table 1).

Reactive oxygen species (ROS) plays a key role in sperm motility. Physiological production at low concentrations has favourable effects on sperm activities and plays a crucial role in sperm metabolism [72]. In the meantime, the excessive production of reactive oxygen species may overwhelm protective mechanisms and cause alterations in lipid and protein layers of the sperm plasma membrane, resulting in lipid damage, protein damage, DNA damage, motility impairment, and alterations in capacity and acrosome reaction [73, 74]. Sperm cell membranes are rich in polyunsaturated fatty acids and vulnerable to oxygen-free radical-induced damage caused by lipid peroxidation [11].

Genis et al. [75] found that a combination of *Lactobacillus* spp., *Bacillus* spp., beer yeast, and photosynthetic bacteria culture which are commercially produced by Chuangbo Modern Natural Agriculture Group (Shanghai, China), has antioxidant properties in response to oxidative stress; they also have a potential action to restore the quality of the sperm damaged by diet stress and show significant decreases in lipid peroxidation and nitric oxide (NO) free radical, and significant increases in superoxide dismutase (SOD) and glutathione peroxidase (GSH-Px).

## 6. Conclusion

Single and multiple strains of lactic acid bacteria supplementation improved steroidogenesis, gametogenesis, and fertility by limiting the invasion of pathogenic bacteria and increasing anti-inflammatory agents, immunological responses, and reproductive hormones.

## Figures and Tables

**Table 1:** Effect of lactic acid bacteria on animal reproduction.

Animals	Strain	Methods	Effects	References
Bovine	*L. rhamnosus* CECT 278, *P. acidilactici* CECT 5915, and *L. reuteri* DSM 20016	Probiotics were administered 1 × 10^8^ CFU/mL in vitro and ex vivo endometrial tissue	Reduced the secretion of inflammation markers (IL-8, IL-1*β*, and IL-6)	[72]
Bovine	*P. acidilactici* CECT 5915, *L*. *rhamnosus* CECT 278, *L. reuteri* DSM 20016, and *L. sakei* DSM 20100	Probiotics were administered 1 × 10^8^ CFU/mL in vitro endometrial tissue	LAB has great potential to modulate endometrial infection and inflammation	[59]
Bovine	*L. sakei* FUA3089, *P. acidilactici* FUA3138, and *P. acidilactici* FUA3140	Probiotic is given intravaginally at a dose of 10^8^–10^9^ CFU	Lowered the incidence of metritis and total uterine infections	[20]
Dairy cow	*L. sakei* FUA3089 as well as *P. acidilactici* FUA3138 and FUA3140	Probiotic is given intravaginally at a dose of 10^10^–10^12^ CFU	Lowered the occurrence of purulent vaginal discharges (PVD)	[60]
Dairy cow	*L. rhamnosus* CECT 278, *P. acidilactici* CECT 5915, and *L. reuteri* DSM 20016	Probiotic is given intravaginally at a dose of 4.5 × 10^10^ CFU	Showed a lower expression of B-defensins and MUC1 in the endometrium	[61]
Swine	*L. acidophilus* and *Kluyveromyces fragilis* (L-4 UCLV)	Probiotic is given orally at a dose of 9 × 10^7^ CFU	Improved health of breeding sows and their offspring	[62]
Chicken	*L. plantarum* SK3494	Probiotic is given orally at a dose of 1.0 × 10^9^ CFU/mL	Improved egg production and performance of laying hens	[63]
Chicken	*Enterococcus faecalis* UGRA10	Probiotic is given orally at a dose of 10^8^	Improved egg production and performance of laying hens	[64]
Chicken	*E*. *faecium* DSM 7134	Probiotic is given orally at a dose of 1.0 × 10^10^ viable spores/g	Increased egg production of laying hens	[65]
Mice	*L*. *rhamnosus* PB01 (DSM-14870)	Probiotic is given orally at a dose of 1 × 10^9^ CFU	Increased serum testosterone, LH, and FSH levels	[49]
Mice	*L*. *plantarum* 2621	Probiotic is given intravaginally at a dose of 10^8^ CFU/20 *µ*l	Protection of the vaginal epithelium	[66]
Mice	*L. acidophilus* ATCC 4356	Probiotic is given orally at a dose of 1 × 10^9^ CFU	Probiotics have antioxidant effects on the testis	[67]
Mice	*L. plantarum* ATCC 8014	Probiotic is given orally at a dose of 10^7^ CFU	Improved testicular kisspeptin and AR expression, Leydig cell count, and effectively increasing epididymal sperm motility and viability	[68]
Mice	*L.rhamnosus* HN001 and *L. acidophilus* GLa-14	Probiotic is given orally at a dose of 0.32–1.28 × 10^11^ CFU	Reduced the number of *Gardnerella vaginalis* detected in the vagina	[69]

## Data Availability

Information about lactic acid bacteria for animal reproduction function were retrieved from a literature search of electronic databases such as the PubMed, Elsevier, Research Gate, Academia, and Google Scholar. The keywords used to perform the search were lactic acid bacteria, animal reproduction, fertility, and infertility. The research data are presented in the table in the article. Supportive data for discussion and comparison were taken from previous studies, which have been cited from recent journals related to the focus of this article. These data are publicly available and accessible online. Detailed sources are provided in References of the manuscript.
